# 7^th^ Brazilian Guideline of Arterial Hypertension: Chapter 1 - Concept, Epidemiology and Primary Prevention

**DOI:** 10.5935/abc.20160151

**Published:** 2016-09

**Authors:** MVB Malachias, FL Plavnik, CA Machado, D Malta, LCN Scala, S Fuchs

## Concept

Arterial hypertension (AH) is a multifactorial clinical condition characterized by sustained elevation of blood pressure (BP) levels ≥ 140 and/or 90 mm Hg. It is often associated with metabolic disorders, functional and/or structural changes in target organs, being worsened by the presence of other risk factors (RF), such as dyslipidemia, abdominal obesity, glucose intolerance and diabetes mellitus (DM).^1,2^ It is independently associated with events such as sudden death, stroke, acute myocardial infarction (AMI), heart failure (HF), peripheral arterial disease (PAD) and fatal and non-fatal chronic kidney disease (CKD).^1-4^

### Medical and social impact of arterial hypertension

North American data from 2015 revealed the presence of AH in 69% of patients on their first episode of AMI, in 77% of those with stroke, in 75% of those with HF and in 60% of those with PAD.^5^ Arterial hypertension accounts for 45% of the cardiac deaths and for 51% of the deaths due to stroke.^6^

### Arterial hypertension and cardiovascular disease in Brazil

In Brazil, AH affects 32.5% (36 million) of the adults, over 60% of the elderly, contributing direct or indirectly to 50% of the deaths due to cardiovascular disease (CVD).^7^ Along with DM, its complications (cardiac, renal and stroke) have high impact on loss of work productivity and on family income, estimated as US$ 4.18 billion from 2006 to 2015.^8^

**Figure 1:**
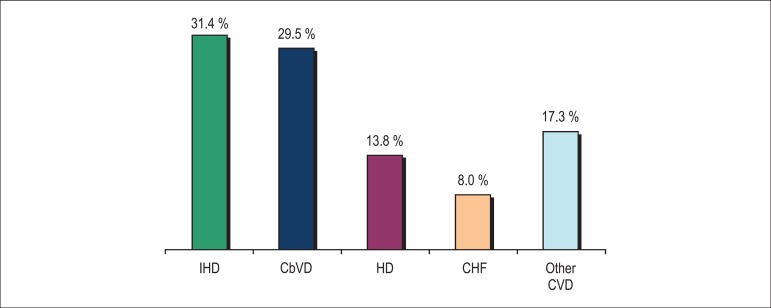
Mortality rate in Brazil due to cardiovascular diseases (CVD) and distribution according to cause in 2013. IHD: ischemic heart disease; CbVD: cerebrovascular disease; HD: hypertensive disease; CHF: congestive heart failure.

In 2013 there were 1,138,670 deaths, 339,672 of which (29.8%) due to CVD, the major cause of death in Brazil (Figure 1).

**Figure 2:**
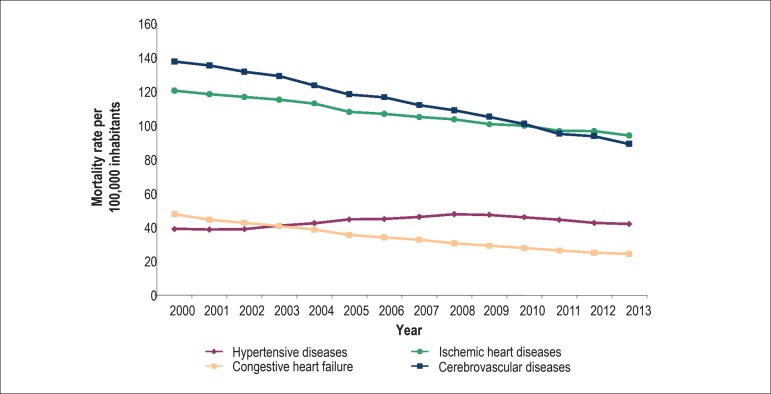
Mortality rate in Brazil due to CVD from 2000 to 2013. Source: Information System on Mortality. Health Surveillance Secretariat, Brazilian Ministry of Health.

The mortality rates have decreased over the years, except for the hypertensive diseases (HD), which increased from 2002 to 2009, showing a reduction trend since 2010. The HD rates in that period ranged from 39/100,000 inhabitants (2000) to 42/100,000 inhabitants. Ischemic heart diseases (IHD) dropped from 120.4/100,000 inhabitants (2000) to 92/100,000 inhabitants (2013), cerebrovascular diseases (CbVD), from 137.7/100,000 inhabitants (2000) to 89/100,000 inhabitants (2013), and congestive HF (CHF), from 47.7/100,000 inhabitants (2000) to 24.3/100,000 inhabitants (2013)^9^ (Figure 2).

In addition, CVD account for the high frequency of hospitalizations, with high socioeconomic costs. Data from the Hospital Information System of the Brazilian Unified Public Health System point to a significant reduction in the hospitalization trend due to AH, from 98.1/100,000 inhabitants (2000) to 44.2/100,000 inhabitants (2013).

**Figure 3:**
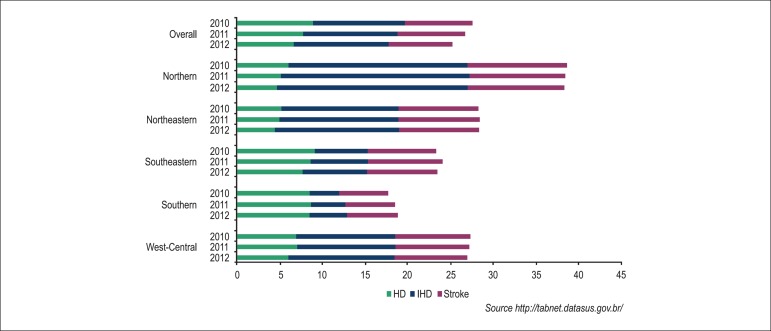
Hospitalization rate in Brazil per 100,000 inhabitants, per geopolitical region, from 2010 to 2012.

Historical hospitalization rates due to CVD by region are shown in Figure 3, with a reduction for HD and stability or reduction trend for stroke, despite the increase in hospitalizations due to IHD.

### Prevalence of arterial hypertension

The prevalence of HA in Brazil varies according to the population studied and the assessment method (Table 1).

**Table 1:** Prevalence of AH according to different approaches

Source	BP	n	General (%)	Men	Women
Picon et al.^10^*	Measured	17,085	28.7 (26.2-31.4)	27.3 (22.5-32.8)	27.7 (23.7-32.0)
Scala et al.^7^	Measured		21.9-46.6	-	-
VIGITEL, 2014**	Self-reported via telephone	40,853	25.0		
PNS, 2013**	Self-reported	62,986	21.4	18.1	21.0
PNS, 2014**	Measured	59,402	22.3	25.3	19.5

BP: blood pressure.

* Meta-analysis; studies from the 2000 decade.

** Note: Self-declared hypertensives under treatment were not considered hypertensive in the VIGITEL and PNS surveys.

In the meta-analysis by Picon et al., the 40 cross-sectional and cohort studies included showed a reduction trend in AH prevalence in the last three decades, from 36.1% to 31.0%.^10^ A study with 15,103 government employees from six Brazilian capitals has reported a 35.8% AH prevalence, with predominance of men (40.1% vs 32.2%).^11^

Data from VIGITEL (2006 to 2014) indicate that the self-reported AH prevalence among individuals aged 18 years and over, living in the capitals, ranged from 23% to 25%, respectively, with no difference in the period assessed, not even regarding sex. The self-reported AH prevalence varied among adults according to age groups as follows: 18 - 29 years, 2.8%; 30 - 59 years, 20.6%; 60 - 64 years, 44.4%; 65 - 74 years, 52.7%; and ≥ 75 years, 55%. The Southeastern region showed the highest self-reported AH prevalence (23.3%), followed by the Southern (22.9%) and West-Central (21.2%) regions. The Northeastern and Northern regions had the lowest rates, 19.4% and 14.5%, respectively.^12^

In 2014, the Brazilian National Health Survey (PNS) measured the BP of selected dwellers from drawn residences, using calibrated digital semi-automated devices. Three BP measurements were taken at two-minute intervals, considering the mean of the last two measurements, inserted in *smartphone*. The overall prevalence of BP ≥140/90 mm Hg was 22.3%, with predominance among men (25.3% *vs* 19.5%), ranging from 26.7% in Rio de Janeiro to 13.2% in Amazonas, with predominance in the urban area as compared to the rural one (21.7% *vs* 19.8%).

### Knowledge, treatment and control

A review^7^ has shown a wide variation of BP knowledge (22% to 77%), treatment (11.4% to 77.5%) and control (10.1% to 35.5%) rates, depending on the population studied (Table 2).

**Table 2:** Blood pressure knowledge, treatment and control in 14 Brazilian population-based studies published from 1995 to 2009.^7^

Author/year per geopolitical region	Place	Number of individuals	Knowledge	Treatment	Control
Southern					
Fuchs et al. 1995	Porto Alegre (RS)	1,091	42.3	11.4	35.5
Gus et al. 2004	Rio Grande Sul	1,063	50.8	40.5	10.4
Oliveira e Nogueira, 2003	Cianorte (PR)	411	63.2	29.9	20.9
Trindade, 1998	Passo Fundo (RS)	206	82.2	53.3	20
Pereira et al. 2007	Tubarão (SC)	707	55.6	50.0	10.1
Southeastern					
Freitas et al. 2001	Catanduva (SP)	688	77	61.8	27.6
Souza et al. 2003	Campos dos Goytacazes (RJ)	1,029	29.9	77.5	35.2
Barreto et al. 2001	Bambuí (MG)	2,314	76.6	62.9	27
Castro et al. 2007	Formiga (MG)	285	85.3	67.3	14.7
Mill et al. 2004	Vitória (ES)	1,656	27.0		
West-Central					
Jardim et al. 2007	Goiânia (GO)	1,739	64.3	43.4	12.9
Cassanelli, 2005	Cuiabá (MT)	1,699	68.3	68.5	16.6
Rosário et al. 2009	Nobres (MT)	1,003	73.5	61.9	24.2
Souza et al. 2007	Campo Grande (MS)	892	69.1	57.3	-

### Prehypertension

**Figure 4:**
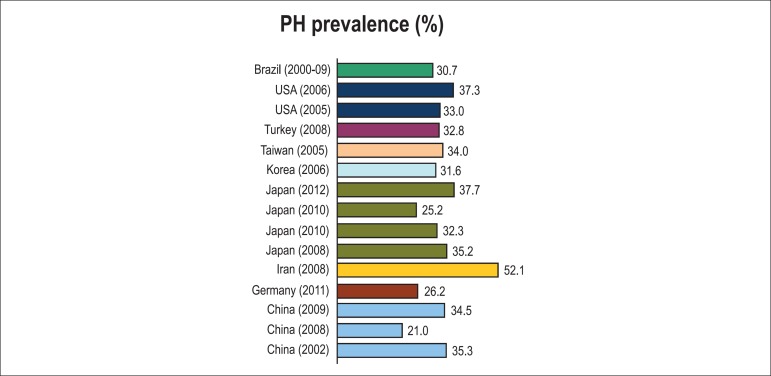
Prevalence of prehypertension (PH).

Prehypertension (PH) is characterized by systolic BP (SBP) between 121 and 139 and/or diastolic BP (DBP) between 81 and 89 mm Hg.^13^ The world prevalence of PH has ranged from 21% to 37,7% in population-based studies, except for Iran (52.1%) (Figure 4).^14^

**Figure 5:**
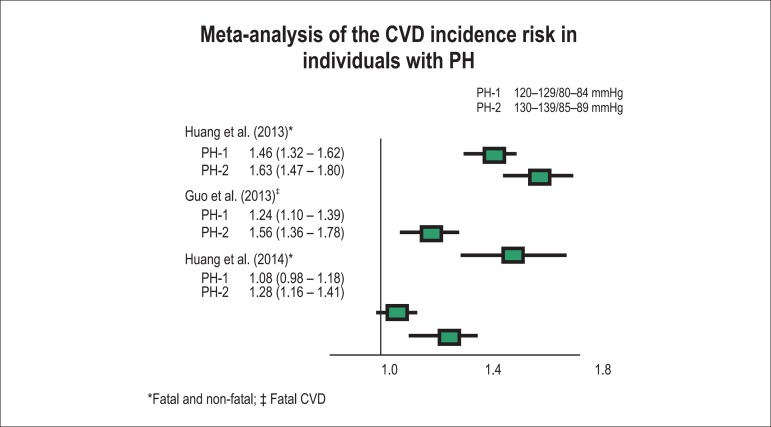
Meta-analysis of the risk of the incidence of cardiovascular disease (CVD) in individuals with prehypertension (PH).

Prehypertension associates with a higher risk of developing AH^15,16^ and cardiac abnormalities.^17^ Approximately one third of the cardiovascular (CV) events attributed to BP elevation occur in individuals with PH.^18^ Meta-analyses of the incidence of CVD, IHD and stroke in prehypertensive individuals have shown a higher risk among those with BP levels between 130 and 139 or 85 and 89 mm Hg than among those with BP levels between 120 and 129 or 80 and 84 mm Hg (Figure 5).^14^

The clinical implication of that epidemiological evidence is that the BP of prehypertensive individuals should be monitored closely, because a significant proportion of them will develop AH and its complications.^2^

### Risk factors for arterial hypertension

#### Age

There is a direct and linear association between aging and AH prevalence related to the increase: i) in life expectancy of the Brazilian population, currently 74.9 years; ii) in the elderly population ≥ 60 years in the past decade (2000 to 2010), from 6.7% to 10.8%.^19^ A meta-analysis of studies performed in Brazil including 13,978 elderly has shown a 68% AH prevalence.^20^

#### Sex and ethnicity

The 2013 Brazilian National Health Survey (PNS) showed a self-reported AH prevalence statistically different between sexes, being higher among women (24.2%) and black individuals (24.2%) as compared to mixed-heritage adults (20.0%), but not white individuals (22.1%). The *Corações do Brasil* Study has reported the following distribution: native population, 11.1%; yellow population, 10%; mixed heritage/mulatto, 26.3%; white, 29.4% and black, 34.8%.^21^ The ELSA-Brazil Study has shown the following prevalences: white, 30.3%; mixed heritage, 38.2%; and black, 49.3%.^11^

#### Overweight and obesity

In Brazil, the 2014 VIGITEL data revealed, between 2006 and 2014, an increase in the prevalence of overweight (BMI ≥ 25 kg/m^2^, 52.5% *vs* 43%. In that same period, obesity (BMI ≥ 30 kg/m^2^ increased from 11.9% to 17.9%, predominating among 35-to-64-year-old individuals and women (18.2% *vs* 17.9%), but remained stable from 2012 to 2014.

#### Salt intake

The excessive consumption of sodium, one of the major RF for AH, associates with CV and renal events.^22,23^

In Brazil, data of the Survey on Family Income (POF), collect from 55,970 dwellings, have shown home availability of 4.7g of sodium/person/day (adjusted for the consumption of 2,000 kcal), exceeding more than twice the maximum recommended consumption (2 g/day), lower in the urban area of the Southeastern region, and higher in the rural area of the Northern region.^24^

The impact of the sodium-rich diet estimated in the 2014 VIGITEL data showed that only 15.5% of the individuals interviewed acknowledged high or extremely high salt content in their meals.^12^

#### Alcohol intake

A chronic and high consumption of alcoholic beverages increases BP consistently. A meta-analysis of 2012, including 16 studies with 33,904 men and 19,372 women compared the consumption intensity between non-drinkers and drinkers.^25^ For women, there was a protective effect with doses lower than 10g of alcohol/day, and risk for AH with a consumption of 30-40g of alcohol/day. For men, the increased risk for AH became consistent from 31g of alcohol/day onwards.

The 2006-2013 VIGITEL data showed that abusive alcohol consumption - at least four doses for women, or at least five doses for men, of alcoholic beverages on the same occasion, within the past 30 days - is stable in the adult population, around 16.4% (24.2% for men and 9.7% for women). For both sexes, abusive alcohol consumption was more often among youngsters, and increased with schooling.^25^

#### Sedentary lifestyle

A population-based study in the city of Cuiabá, Mato Grosso State, (n = 1,298 adults ≥ 18 years) has revealed a 75.8% overall prevalence of sedentary lifestyle (33.6% during leisure time; 19.9% at work; 22.3% during both). A significant association of AH was observed with age, male sex, overweight, central adiposity, sedentary lifestyle during leisure time and work, less than 8 years of schooling, *per capita* income < 3 minimum wages.^26^

Brazilian National Health Survey (PNS) data indicate that insufficiently active individuals (adults not practicing at least 150 minutes per week of physical activity including leisure, work and displacement time) represent 46.0% of the adults, the percentage being significantly higher among women (51.5%). The frequencies of insufficiently active individuals differed between age groups, mainly among the elderly (62.7%) and the adults with no formal education and those with incomplete elementary education (50.6%).^27^

#### Socioeconomic factors

Adults with lower schooling (no formal education or incomplete elementary education) have a higher prevalence of self-reported AH (31.1%). The proportion decreases among those with complete elementary education (16.7%), being 18.2% among those with complete higher education.^26^ However, the ELSA Brazil Study, performed with employees of six Brazilian universities and university-affiliated hospitals with higher schooling, has shown a 35.8% AH prevalence, higher among men.^11^

#### Genetics

Brazilian studies assessing the impact of genetic polymorphisms in the *quilombola* population could not identify a more prevalent pattern, showing the strong impact of miscegenation, and hindering the identification of a genetic pattern for the elevation of BP levels.^28,29^

### Strategies for the implementation of preventive measures

The strategies for preventing the development of AH comprise public policies for health in combination with action from the medical societies and communication media. They should be aimed at stimulating early diagnosis, continuous treatment, control of BP and associated RF, by use of lifestyle changes and/or regular use of medications.
